# Safety and efficacy of tislelizumab plus chemotherapy as preoperative treatment in potentially resectable locally advanced non-small-cell lung cancer patients

**DOI:** 10.1093/icvts/ivad157

**Published:** 2023-09-19

**Authors:** Xuhua Huang, Linhai Zhu, Jiacong Liu, Yanye Wang, Li Yu, Simeng Wu, Wang Lv, Jian Hu

**Affiliations:** Department of Thoracic Surgery, The First Affiliated Hospital, Zhejiang University School of Medicine, Hangzhou, China; Department of Thoracic Surgery, The First Affiliated Hospital, Zhejiang University School of Medicine, Hangzhou, China; Department of Thoracic Surgery, The First Affiliated Hospital, Zhejiang University School of Medicine, Hangzhou, China; Department of Thoracic Surgery, The First Affiliated Hospital, Zhejiang University School of Medicine, Hangzhou, China; Department of Thoracic Surgery, The First Affiliated Hospital, Zhejiang University School of Medicine, Hangzhou, China; Department of Cardiothoracic Surgery, The Shaoxing Second Hospital, Shaoxing, China; Department of Thoracic Surgery, The First Affiliated Hospital, Zhejiang University School of Medicine, Hangzhou, China; Department of Thoracic Surgery, The First Affiliated Hospital, Zhejiang University School of Medicine, Hangzhou, China; Key Laboratory of Clinical Evaluation Technology for Medical Device of Zhejiang Province, Hangzhou, China

**Keywords:** Tislelizumab, Chemotherapy, Efficacy, Safety, Non-small-cell lung cancer

## Abstract

**OBJECTIVES:**

Locally advanced non-small-cell lung cancer (LA-NSCLC) requires more preoperative regiments in the era of immunotherapy. Tislelizumab was approved for first-line treatment for advanced lung cancer, bringing hope for preoperative therapy in LA-NSCLC. The aim of this study was to investigate the safety and efficacy of preoperative tislelizumab plus chemotherapy in LA-NSCLC.

**METHODS:**

The medical records at the First Affiliated Hospital of Zhejiang University were examined retrospectively from September 2019 to June 2022 for this descriptive single-arm cohort study. Patients with LA-NSCLC were treated with tislelizumab plus platinum-based dual-drug regimens for 2–6 cycles and regular imaging assessments were performed every 1–2 cycles. Data including demographic characteristics, clinicopathological staging, adverse events and surgery-related details were recorded in specifically designed forms.

**RESULTS:**

Forty patients met the inclusion criteria of the study and 23 patients underwent curative intent surgeries. Significantly clinical and pathological downstaging was observed, with the objective response rate being 65.00%, leading to a major pathological remission (MPR) rate of 56.52% and a pathological complete remission (pCR) rate of 34.78%. Grade 3–4 treatment-related adverse events occurred in 4 patients and no perioperative death occurred. The 1-year progress-free survival rate and the 1-year overall survival rate were 85.0% and 90.0%, respectively.

**CONCLUSIONS:**

Tislelizumab plus chemotherapy as preoperative therapy demonstrates promising antitumour activity for potentially resectable LA-NSCLC with high MPR, pCR and acceptable toxicity and survival.

**Figure:**
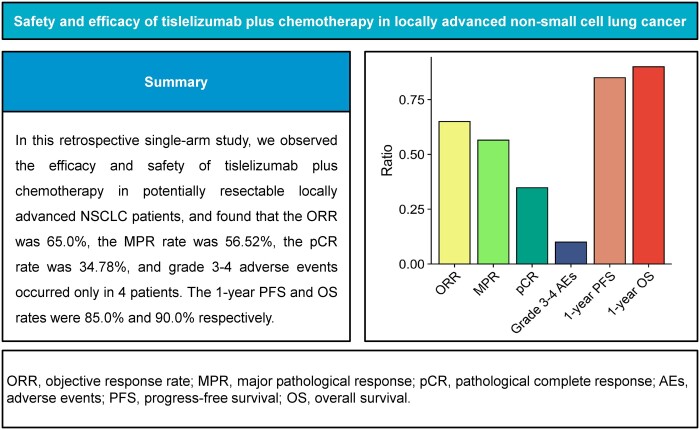

## INTRODUCTION

Non-small-cell lung cancer (NSCLC) accounts for ∼85% of lung cancer [1]. Still, only about 25% of NSCLC cases can be diagnosed at an early stage [2], resulting in that lung cancer remaining the leading cause of cancer-related mortality [3]. With the progress of drug research and the shift of treatment paradigm, more and more locally advanced patients have the opportunity to undergo surgical treatment after preoperative therapy or conversion therapy.

Preoperative chemotherapy had shown a slight benefit in NSCLC [2]. Several randomized controlled trials have confirmed better results of preoperative immunotherapy plus chemotherapy than chemotherapy alone in NSCLC, such as Checkmate 816 [4] and NADIM [5] for nivolumab, SAKK16/14 [6] for durvalumab and NCT02716038 [7] for atezolizumab.

As a programmed cell death protein-1 (PD-1) inhibitor, tislelizumab is an innovative humanized IgG4 monoclonal antibody developed by BeiGene (Beijing, China) [8]. Tislelizumab has been studied in advanced lung cancer. Results from a clinical phase II study [9] showed that Tislelizumab combined with chemotherapy showed encouraging antitumour activity and was generally well tolerated. In phase III trials, RATIONAL-303 [10], RATIONAL-304 [11] and RATIONAL-307 [12] trials investigated tislelizumab plus different chemotherapy regimens in late-line or first-line treatment of advanced NSCLC patients, respectively, and the results showed that patients had survival benefits and improved quality of life [13]. Some studies have also combined tislelizumab with agents of other mechanisms in advanced lung cancer, such as anlotinib [14], but further studies are needed.

The application experience of tislelizumab in advanced NSCLC provides new treatment strategies for locally advanced or relatively early-stage tumours. Tislelizumab plus chemotherapy as a preoperative therapy showed good antitumour activity in resectable oesophageal squamous cell carcinoma with high major pathological remission (MPR) rate, pathological complete remission (pCR) rate, R0 rate and acceptable toxicity [15–17]. In some case reports, specific advanced thoracic solid tumours showed MPR or even pCR after receiving tislelizumab (single agent or combined with chemotherapy), such as lung squamous cell carcinoma [18] and pancoast tumour [19]. This suggests that the preoperative use of tislelizumab in potentially resectable locally advanced NSCLC (LA-NSCLC) may provide additional benefits. However, there is no systematic analysis of the application of preoperative tislelizumab plus chemotherapy in LA-NSCLC.

In view of the heterogeneity and treatment complexity of NSCLC, the use of tislelizumab provides more possibilities for the preoperative treatment of NSCLC. This retrospective study examined the efficacy and safety of tislelizumab combined with chemotherapy in the preoperative setting of potentially resectable LA-NSCLC.

## PATIENTS AND METHODS

### Ethics statement

The Clinical Research Ethics Committee of the First Affiliated Hospital of Zhejiang University School of Medicine (FAHZU) approved the study (Grant No. 2021 IIT No. 844), and the written informed consent was obtained before all the treatments from the patients.

### Patients’ enrolment

This study was designed as a retrospective single-arm cohort study. The primary end point of this study was objective response rate (ORR). Based on previous research on preoperative ICIs plus chemotherapy in NSCLC [20], the null hypothesis (H0) was set at an ORR of 10% or lower, while the alternative hypothesis (H1) was set at 40%. To determine the appropriate sample size, R. P. A' Hern's sample size table [21] was used, with one-sided α = 0.01 and β = 0.9. Consequently, a sample size of 24 was deemed theoretically sufficient.

The study examined the entire cohort of patients who had undergone the administration of preoperative tislelizumab plus chemotherapy at the Department of Thoracic Surgery, FAHZU, between September 2019 and June 2022. Patient data including demographic information, blood test results, imaging data and surgical details were recorded in pre-designed tables, extracted from the hospital's digital medical record system.

The eligibility criteria include: (i) age of 18 years or older; (ii) histopathologically confirmed NSCLC before medication; (iii) TNM stage II–III; (iv) Eastern Cooperative Oncology Group—Performance Status (ECOG-PS) score of less 2; and (v) clinical assessment by the medical team indicating the potential resectability of the tumour or anticipated benefit from preoperative treatment.

The exclusion criteria were: (i) absence of premedication chest imaging data [either computed tomography (CT) or positron emission tomography–computed tomography] or having only 1 imaging data in the whole following-up period; (ii) prior receipt of chemotherapy, radiotherapy, interventional therapy, targeted therapy, alternative immunotherapy or surgical intervention; (iii) concurrent active pulmonary tuberculosis, hepatitis C infection or autoimmune disease; (iv) presence of severe ventilatory disorder or severe gas exchange abnormalities; (v) demonstrated immunosuppression or deficiency, such as ongoing steroid therapy or confirmed HIV infection; (vi) documented allergic reactions or intolerance to any component of tislelizumab or chemotherapy agents; (vii) inclusion in other clinical trials; and (viii) previous genetic analysis identifying targetable gene mutations, including but not limited to epidermal growth factor receptor (EGFR), Kirsten rats arcomaviral oncogene homolog (KRAS), neuroblastoma RAS viral oncogene homolog (NRAS), B-rapidly accelerated fibrosarcoma protein (BRAF), human epidermal growth factor receptor-2 (HER-2), Mesenchymal-epithelial transition factor (MET), Phosphatidylinositol-4,5-bisphosphate 3-kinase catalytic subunit alpha (PIK3CA), Anaplastic lymphoma kinase (ALK), C-ros oncogene 1 (ROS1) and REarranged during Transfection gene (RET).

Patients underwent a comprehensive evaluation before receiving all preoperative treatments, including chest CT, brain magnetic resonance imaging, pulmonary function testing, blood routine test and serum biochemical analysis and abdominal B-ultrasound. Positron emission tomography–computed tomography assessment was assigned for those with a higher risk of metastasis. To clarify the pathological types, bronchoscopy was routinely performed, and lung biopsy was performed for the patients with peripheral lung masses identified through CT imaging.

The follow-up continued for at least 1 year after patients received treatment. Progression-free survival (PFS) was defined as the time from treatment initiation until the date of disease progression or death from any cause. Overall survival (OS) was defined as the time from treatment initiation to the date of death from any cause.

### Preoperative therapy procedures

The patients who participated in the study were scheduled to receive intravenous treatment consisting of tislelizumab (200 mg) plus chemotherapy for 2–6 cycles before undergoing surgical resection. Each cycle lasted for 21 days, and the chemotherapy regimens were platinum-based 2-drug combinations. Patients with histopathologically diagnosed lung squamous cell carcinoma (LUSC) received nab-paclitaxel (260 mg/m^2^) plus carboplatin (AUC = 5), while those diagnosed with lung adenocarcinoma (LUAD) were given pemetrexed (500 mg/m^2^) plus carboplatin (AUC = 5). The treatment regimen for each patient was determined by the investigator based on a combination of standard criteria and their individual experience with patients' reactions.

### Tumour response evaluation

The patients underwent chest CT scans every 2 cycles until surgical resection. Tumour staging was evaluated based on the 8th edition of AJCC TNM staging at baseline (clinical staging), after preoperative therapy (yield clinical staging), and after surgical pathological reaffirmation (yield pathological staging). The Response Evaluation Criteria in Solid Tumour version 1.1 was used to assess the tumour treatment response in the target lesions [22]. Complete response (CR) was defined as the disappearance of all target lesions, while partial remission (PR) was defined as a reduction in the overall diameter of the target lesions by at least 30%. Stable disease was defined as a change in the target lesion between −30% and 20%, with no new lesions formed, and progressive disease was defined as an increase in the overall diameter of target lesions by at least 20%, or the development of new lesions.

### Treatment-related adverse events evaluation

To evaluate adverse events (AEs), the study employed the Common Terminology Criteria for Adverse Events V.5.0. Regular blood routine tests and serum biochemical tests were used to evaluate blood system disorders, endocrine disorders, hepatic function and renal function. Patients' complaints were used to evaluate gastrointestinal reactions, sensory neural disorders and skin reactions.

### Surgical treatment procedures

The medical team reassessed patients for surgical indications within 4–6 weeks after completing preoperative therapy. The patients in this study underwent open surgery and video-assisted thoracoscopic surgery, which included lung lobectomy, lung sleeve resection and pneumonectomy, along with standardized lymph node dissection. The surgical approach was determined based on preoperative CT images, but adjustments were made as necessary during the surgery, depending on factors such as dense adhesions or complex anatomical structures.

### Pathological reaffirmation

The surgical specimens were preserved in 4% neutral formaldehyde, sectioned and embedded in paraffin. Then, the samples were sliced and stained with standard haematoxylin–eosin, and some specimens underwent immunohistochemical examination to determine tumour pathology. Pathological results, including pathological type, degree of differentiation, depth of invasion, resection margin, lymph node metastasis and degree of tumour regression were collected by 2 independent investigators based on the pathology report and pathological photographs. Pathological complete response (pCR) was defined as the absence of cancer cells, determined by estimating the proportion of remaining viable tumour cells in the original lesion area, while major pathological response (MPR) was defined as <10% residual viable cancer cells.

### Statistical analysis

Continuous variables were represented as mean ± standard deviation for normal distributed data or median (range) for skewed distributed data. Categorical variables were represented as the number of cases and percentages. To explore the possible factors associated with disease remission, the population was stratified according to imaging assessment whether the population was in remission or not. Those with CR or PR in imaging assessment were classified as the remission group, while those with stable disease or progressive disease were classified as the non-remission group.

Independent Student’s *t*-tests were applied for continuous variables, and Pearson Chi-square tests or Fisher’s exact tests were applied for categorical variables in demographic and clinical characteristics analysis. Paired samples Wilcoxon signed rank tests were applied in staging change analysis. Binary logistic regression model was applied to calculate odds ratios and corresponding 95% confidence intervals for possible factors affecting lesion imaging remission.

SPSS software (IBM, version 25.0) was used to perform all the analyses. A two-sided *P*-value of <0.05 was considered significant. Visualizations were performed using the ggplot2 R package (version 3.3.6) in R software (version 4.2.1).

## RESULTS

### Baseline demographic characteristics

**Figure 1:**
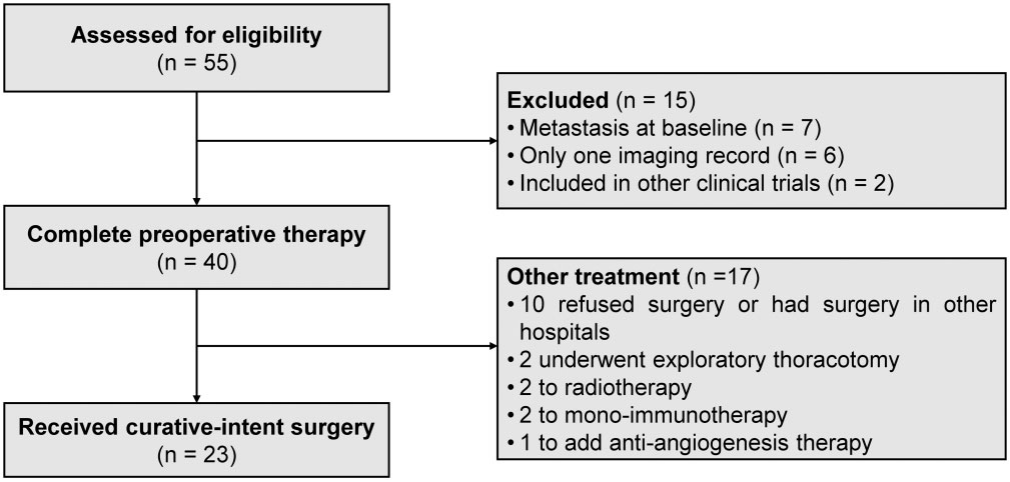
Schematic workflow of patient enrolment and following treatments.

The patient enrolment and detailed treatment profiles are summarized in Fig. 1. This study collected 40 patients for final analysis. Table 1 summarizes the demographic and clinical characteristics of the patients in this study at baseline. From September 2019 to June 2022, 55 individuals were collected, and 40 patients were included for final analysis, among which the average age was 67.95 ± 7.00 years old. Males accounted for 95.00% (38/40) and smokers accounted for 67.50% (27/40). Hypertension was the most common comorbidities in the patients [42.50% (17/40)]. The tumours were located in all anatomical lobes of the lung, but most were in the superior lobe of the left lung (30.00%). Preoperative pathological examination confirmed that LUSC accounted for 85.00% (34/40) and LUAD accounted for 15.00% (6/40). Binary logistic regression including all variables collected at baseline showed that age was the only factor that significantly associated with remission (odds ratio: 1.146, 95% confidence interval: 1.011–1.299), indicating that the risk of non-remission after tislelizumab plus chemotherapy increased 14.6% as each age unit increase.

**Table 1:** Characteristics of the patients treated with tislelizumab plus chemotherapy at baseline

Characteristic	All (*n* = 40)	Remission group (*n* = 26)	Non-remission group (*n* = 14)	Statistical value	*P*-Value
Age, mean (SD)	67.95 (7.00)	66.08 (5.84)	71.43 (7.84)	−2.449	0.019
Gender (male), *n* (%)	38 (95.00)	25 (96.15)	13 (92.86)	0.208	1.000
ECOG performance status (=0), *n* (%)	25 (62.50)	18 (69.23)	7 (50.00)	1.436	0.310
Smoking status (yes), *n* (%)	27 (67.50)	19 (73.07)	8 (57.14)	1.053	0.480
Drinking status (yes), *n* (%)	16 (40.00)	9 (34.62)	7 (50.00)	0.897	0.500
Comorbidities, *n* (%)					
Pulmonary disease	11 (27.50)	7 (26.92)	4 (28.57)	0.012	1.000
Cardiac disease	6 (15.00)	5 (19.23)	1 (7.14)	1.043	0.399
Kidney disease	0 (0.00)	0 (0.00)	0 (0.00)	NA	NA
Diabetes mellitus	1 (2.50)	0 (0.00)	1 (7.14)	1.905	0.350
Hypertension	17 (42.50)	10 (38.46)	7 (50.00)	0.496	0.521
Pathology, *n* (%)				3.801	0.074
LUSC	34 (85.00)	20 (76.92)	14 (100.00)		
LUAD	6 (15.00)	6 (23.08)	0 (0.00)		
Tumour location, *n* (%)				0.114	0.064
Superior lobe of left lung	12 (30.00)	9 (34.62)	3 (21.43)		
Inferior lobe of left lung	5 (12.50)	3 (11.54)	2 (14.29)		
Superior lobe of right lung	10 (25.00)	6 (23.07)	4 (28.57)		
Middle lobe of right lung	1 (2.50)	0 (0.00)	1 (7.14)		
Inferior lobe of right lung	7 (17.50)	5 (19.23)	2 (14.29)		
Hilum of left lung	5 (12.50)	3 (11.54)	2 (14.29)		
Treatment cycle, *n* (%)				−0.756	0.528
2	9 (22.50)	4 (15.38)	5 (35.71)		
3	3 (7.50)	3 (11.54)	0 (0.00)		
4	25 (62.50)	17 (65.38)	8 (57.14)		
6	3 (7.50)	2 (7.69)	1 (7.14)		
Baseline tumour size, mean (SD)	50.47 (18.94)	48.42 (17.00)	54.26 (22.28)	−0.927	0.360

ECOG: Eastern Cooperative Oncology Group; LUAD: lung adenocarcinoma; LUSC: lung squamous cell cancer; SD: standard deviation.

### Perioperative therapeutic and surgical efficacy

**Figure 2:**
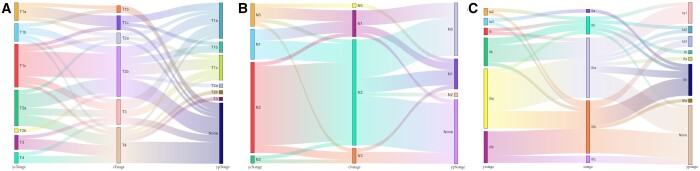
Sankey diagram demonstrating overall clinical and pathological downstaging of all patients compared between the central column (cstage) and the left column (ycstage) or the right column (ypstage). (**A**) T downstaging; (**B**) N downstaging; and (**C**) overall downstaging.

In accordance with Response Evaluation Criteria in Solid Tumour version 1.1, the preoperative imaging assessment showed 26 patients achieved PR or CR, implying an ORR of 65.0%. Clinical and pathological downstaging details were present in Fig. 2. Significant T downstaging (*Z* = −4.97, *P* < 0.001), N downstaging (*Z* = −2.68, *P* = 0.007) and overall downstaging (*Z* = −3.78, *P* < 0.001) were observed among all enrolled patients (Supplementary Material, Table S1). After preoperative therapy, the maximum lesion diameter was significantly decreased (−16.95%, *t* = −5.47, *P* < 0.001) (Supplementary Material, Fig. S1). Besides, postoperative pathology showed that the clinical stage decreased significantly in T stage (*Z* = −4.22, *P* < 0.001), N stage (*Z* = −3.85, *P* < 0.001) and overall stage (*Z* = −4.05, *P* < 0.001) (Supplementary Material, Table S2).

Surgical efficacy is summarized in Table 2. Twenty-three patients underwent curative intent surgeries. Ten patients underwent open surgery, with lobectomy being the most prevalent operation (10/23, 43.48%). The average operation duration was 171.0 ± 73.5 min, with a median estimated blood loss of 50 (10–1500) ml. The R0 resection rate was 100%. The average number of lymph nodes dissected during surgery was 19.96 ± 9.41. Postoperative pathology revealed pCR in 8 (34.78%) and MPR in 13 (56.52%) of the patients treated with surgery.

**Table 2:** Outcomes of non-small-cell lung cancer patients undergoing curative intent surgery (*n* = 23)

Outcomes	All (*n* = 23)
Time from first preoperative therapy to surgery (days), median (range)	94 (28–113)
Operation time (min), mean (SD)	171.0 (73.5)
Estimated blood loss (ml), median (range)	50 (10–1500)
Total number of lymph node dissections during surgery, mean (SD)	19.96 (9.41)
R0 resection, *n* (%)	23 (100.00)
Length of hospital stay (days), mean (SD)	13.5 (6.8)
Entry method, *n* (%)	
VATS	7 (30.43)
Conversion	6 (26.09)
Open	10 (43.48)
Operation method, *n* (%)	
Lobectomy	10 (43.48)
Sleeve resection	9 (39.13)
Pneumonectomy	4 (17.39)
Differentiation grade, *n* (%)	
2	3 (13.04)
3	7 (30.43)
NA	13 (56.52)
Pathological response, *n* (%)	
Viable cells = 0%	8 (34.78)
Viable cells ≤ 10%	5 (21.74)
Viable cells > 10%	10 (43.48)
Postoperative complication, *n* (%)	1 (4.35)

SD: standard deviation; VATS: video-assisted thoracoscopic surgery.

### Preoperative treatment-related adverse events and surgery-related complications

Treatment-related adverse events (trAEs) of preoperative therapy are summarized in Table 3. During the preoperative therapy process, most patients suffered grade 1–2 tolerable trAEs. Four patients (10.0%) experienced grade 3–4 AEs (including leucocyte suppression, agranulocytosis, anaemia and skin reaction). Haematological AEs were the most common treatment-related AEs, in descending order of frequency: anaemia, leucocyte suppression, agranulocytosis, coagulation disorders and thrombocytopaenia. Only 1 patient suffered from grade 3 postoperative complication. The patient experienced progressive haemothorax, which was controlled after a second operation.

**Table 3:** Treatment-related adverse events of preoperative tislelizumab plus chemotherapy (*n* = 39)

trAEs, *n* (%)	None	Grade 1	Grade 2	Grade 3	Grade 4
Haematological events					
Leucocyte suppression	18 (46.15)	14 (35.9)	6 (15.38)	1 (2.56)	0 (0.00)
Agranulocytosis	32 (82.05)	4 (10.26)	2 (5.13)	1 (2.56)	0 (0.00)
Anaemia	13 (33.33)	19 (48.72)	5 (12.82)	2 (5.13)	0 (0.00)
Thrombocytopaenia	38 (97.44)	1 (2.56)	0 (0.00)	0 (0.00)	0 (0.00)
Coagulation disorders	37 (94.87)	2 (5.13)	0 (0.00)	0 (0.00)	0 (0.00)
Gastrointestinal events					
Nausea	38 (97.44)	1 (2.56)	0 (0.00)	0 (0.00)	0 (0.00)
Vomiting	34 (87.18)	5 (12.82)	0 (0.00)	0 (0.00)	0 (0.00)
Diarrhea	39 (100.00)	0 (0.00)	0 (0.00)	0 (0.00)	0 (0.00)
Constipation	29 (74.36)	6 (15.38)	4 (10.26)	0 (0.00)	0 (0.00)
Myocarditis	39 (100.00)	0 (0.00)	0 (0.00)	0 (0.00)	0 (0.00)
Pneumonia	37 (94.87)	2 (5.13)	0 (0.00)	0 (0.00)	0 (0.00)
Liver damage	27 (69.23)	10 (25.64)	2 (5.13)	0 (0.00)	0 (0.00)
Renal insufficiency	38 (97.44)	1 (2.56)	0 (0.00)	0 (0.00)	0 (0.00)
Skin reaction	29 (74.36)	5 (12.82)	4 (10.26)	1 (2.56)	0 (0.00)
Hypothyroidism	38 (97.44)	0 (0.00)	1 (2.56)	0 (0.00)	0 (0.00)
Sensory neurotoxicity	36 (92.31)	2 (5.13)	1 (2.56)	0 (0.00)	0 (0.00)

trAEs: treatment-related adverse events.

### Survival outcomes at least 1 year after surgery

**Figure 3:**
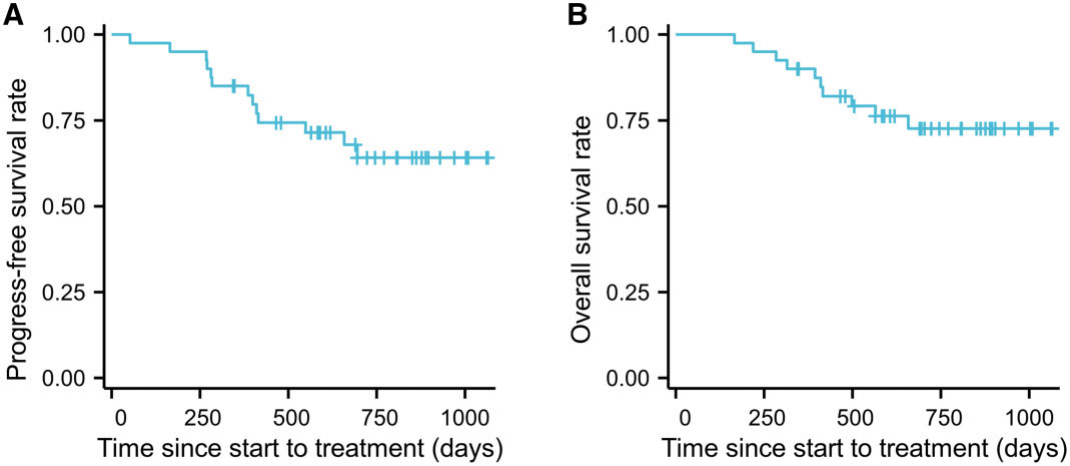
Progress-free survival and overall survival. (**A**) Kaplan–Meier curves of progression-free survival for all patients. (**B**) Kaplan–Meier curves of overall survival for all patients.

At the end of the study period, neither median PFS nor median OS had been reached. The 1-year PFS rate was 85.0% (Fig. 3A) and the 1-year OS rate was 90.0% (Fig. 3B).

## DISCUSSION

Despite progress in treatment modalities, NSCLC still remains a leading cause of cancer-related mortality, highlighting the urgent need for continued research and improved screening efforts to detect and treat in early stages. Although the efficacy of tislelizumab plus chemotherapy after disease progression has been demonstrated, first-line preoperative immunotherapy in NSCLC remains inadequately understood.

This study investigated the efficacy and safety of preoperative tislelizumab in combination with chemotherapy in potentially resectable NSCLC, providing new insights and evidence for the use of tislelizumab in preoperative therapy for NSCLC.

The results of these studies showed promising efficacy and acceptable toxicity of tislelizumab in combination with chemotherapy in solid tumours. In the preoperative therapy design, a retrospective cohort study [23] involved 8 LUSC patients receiving tislelizumab plus chemotherapy, of whom 6 achieved MPR and 2 achieved pCR. Our research results are similar to the results of the previous study. However, the larger sample size of our study verifies and provides more convincing evidence.

In this study, all LUAD patients showed at least PR, but the majority of the included patients were LUSC. Peripheral lung cancer (mainly LUAD) may undergo surgery at the time of initial diagnosis, while difficult central lung cancer (mainly LUSC) tends to receive preoperative treatment. There is few research investigating the histopathological advantage of neoadjuvant immunotherapy plus chemotherapy in NSCLC, and the different response may be attributed to the highly heterogeneous immune landscape [24].

Although there was no statistical difference in the proportion of pathological response in the different imaging response groups in the study, the inconsistency between imaging and pathological evaluation suggests that, although the lesion regression was not ideal on imaging, surgical strategies could be adopted more actively and more benefits could be achieved in pathological CR.

The trAEs of tislelizumab application in lung cancer exhibited considerable diversity, with the most prevalent being blood system disorders, a pattern that aligns with our study findings. Other case reports include membranous nephropathy [25], colitis [26], enteritis [27] and multiple organ damage [28]. In contrast to pembrolizumab and nivolumab, it attaches to PD-1 at a distinct epitope, with a dissociation rate for PD-1 that is ∼100- and 50-fold slower, respectively [29]. The drug's low affinity to FcγRI on macrophages is responsible for eliminating antibody-dependent phagocytosis and boosting its antitumour effects compared to other immune drugs [8]. It is still necessary to pay attention to the possibility of these AEs in clinical application and give corresponding measures in time.

### Limitations

However, this study is not without its limitations. First, this was a retrospective study and was able to provide a potentially insufficient level of evidence. Second, this study did not find a benefit population of preoperative tislelizumab plus chemotherapy in NSCLC by stratified analysis, which may be credited to the limited number of patients included. Additionally, owing to constraints intrinsic to real-world clinical practice, the utilization of further molecular markers for the establishment of correlations or the development of predictive models remains unattainable.

## CONCLUSION

Tislelizumab plus chemotherapy as preoperative therapy demonstrates promising antitumour activity for locally advanced NSCLC with high MPR, pCR and R0 resection rates and acceptable tolerability. As far as we know, this is the first study focusing on the efficacy and safety of preoperative tislelizumab plus chemotherapy in NSCLC and this study provides evidence for surgery. More powerful, prospective randomized controlled trials are needed to evaluate its effect in the future.

## Supplementary Material

ivad157_Supplementary_Data

## Data Availability

The data underlying this article will be shared on reasonable request to the corresponding author.

## ABBREVIATIONS

AEs: Adverse events
CR: Complete response
CT: Computed tomography
LA-NSCLC: Locally advanced non-small-cell lung cancer
LUAD: Lung adenocarcinoma
LUSC: Lung squamous cell carcinoma
NSCLC: Non-small-cell lung cancer
ORR: Objective response rate
OS: Overall survival
PD-1: Programmed cell death protein-1
PFS: Progression-free survival
PR: Partial remission
trAEs: Treatment-related adverse events
