# Supplementary material for: Safety and efficacy of tislelizumab plus chemotherapy as preoperative treatment in potentially resectable locally advanced non-small-cell lung cancer patients
Source: Interdiscip Cardiovasc Thorac Surg. 2023 Sep 19;38(1):ivad157. doi: 10.1093/icvts/ivad157 (PMC10761202; doi:10.1093/icvts/ivad157)
Supplement: ivad157_Supplementary_Data [file ivad157_supplementary_data.docx]

**FIGURE LEGENDS**

**Supplementary Figure 1.**

Maximum lesion diameter change (%) before preoperative therapy and after preoperative therapy.


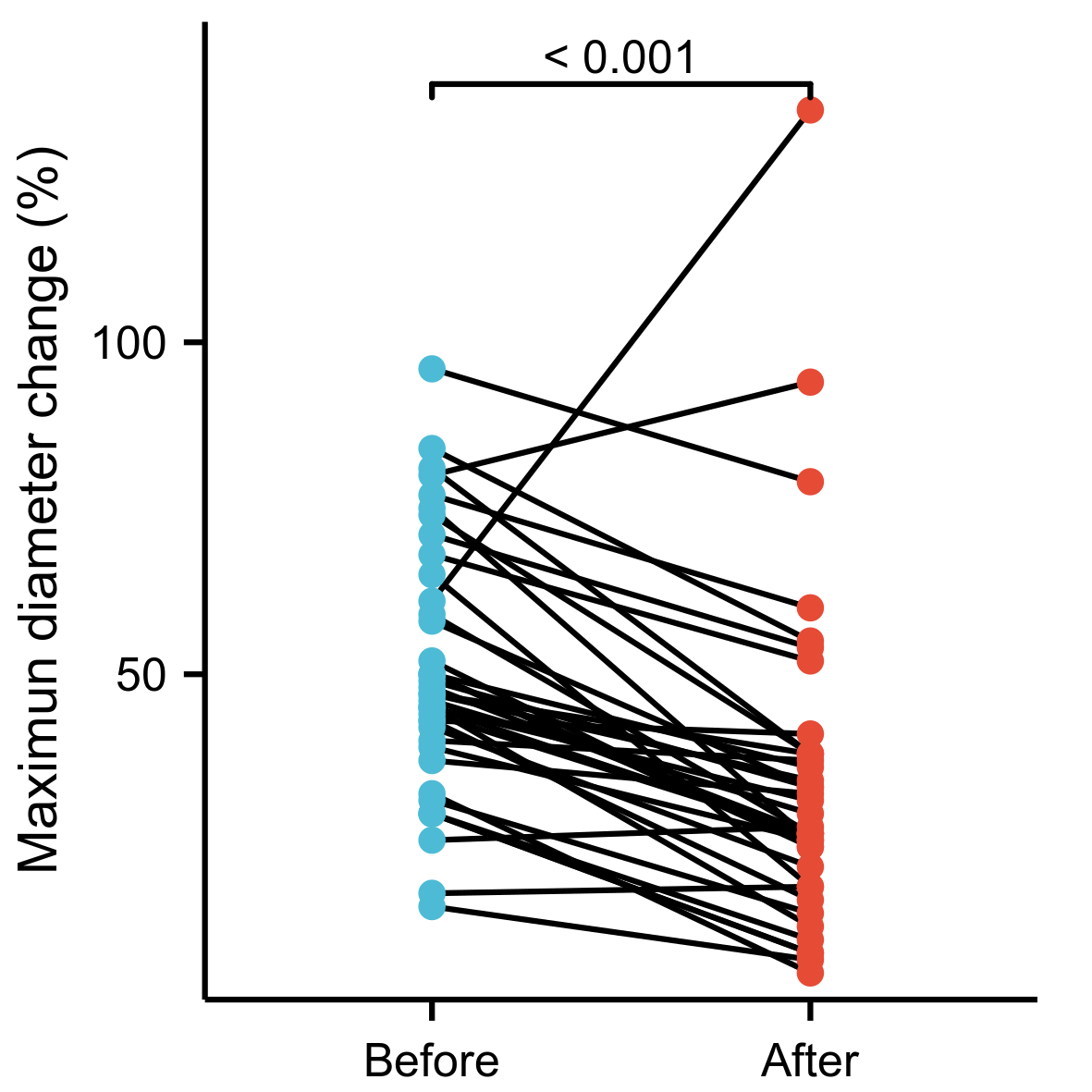


**Supplementary Figure 1.**

**Supplementary Table 1.** Changes of clinical stage of potentially resectable NSCLC patients before (cstage) and after (ycstage) preoperative treatment (n = 40).

| **Characteristic** | **cstage** | **ycstage** | **Z value** | **p-value** |
| --- | --- | --- | --- | --- |
| **T stages, n (%)** |  |  | -4.97 | <0.001 |
| 1a | 0 (0) | 5 (12.5) |  |  |
| 1b | 2 (5.0) | 5 (12.5) |  |  |
| 1c | 4 (10.0) | 12 (30.0) |  |  |
| 2a | 3 (7.5) | 10 (25.0) |  |  |
| 2b | 14 (35.0) | 1 (2.5) |  |  |
| 3 | 7 (17.5) | 4 (10.0) |  |  |
| 4 | 10 (25.0) | 3 (7.5) |  |  |
| **N stage, n (%)** |  |  | -2.68 | 0.010 |
| 0 | 1 (2.5) | 6 (15.0) |  |  |
| 1 | 7 (17.5) | 8 (20.0) |  |  |
| 2 | 28 (70.0) | 24 (60.0) |  |  |
| 3 | 4 (10.0) | 2 (5.0) |  |  |
| **Clinical stage, n (%)** | |  | -3.78 | <0.001 |
| Ia2 | 0 (0) | 2 (5.0) |  |  |
| Ia3 | 0 (0) | 2 (5.0) |  |  |
| Ib | 0 (0) | 2 (5.0) |  |  |
| IIa | 1 (2.5) | 0 (0) |  |  |
| IIb | 5 (12.5) | 8 (20.0) |  |  |
| IIIa | 17 (42.5) | 17 (42.5) |  |  |
| IIIb | 15 (37.5) | 9 (22.5) |  |  |
| IIIc | 2 (5.0) | 0 (0) |  |  |

**Supplementary Table 2.** Changes of stage of NSCLC patients before (cstage) and after surgery (ypstage) (n = 23).

| **Characteristic** | **cstage** | **ypstage** | **Z value** | **p-value** |
| --- | --- | --- | --- | --- |
| **T stages, n (%)** |  |  |  |  |
| 1a | 0 (0.00) | 10 (43.48) | -4.217 | <0.001 |
| 1b | 0 (0.00) | 3 (13.04) |  |  |
| 1c | 3 (13.04) | 7 (30.43) |  |  |
| 2a | 2 (8.70) | 1 (4.35) |  |  |
| 2b | 9 (39.13) | 1 (4.35) |  |  |
| 3 | 5 (21.74) | 1 (4.35) |  |  |
| 4 | 4 (17.39) | 0 (0.00) |  |  |
| **N stage, n (%)** |  |  | -3.849 | <0.001 |
| 0 | 1 (4.35) | 14 (60.87) |  |  |
| 1 | 6 (26.09) | 8 (34.78) |  |  |
| 2 | 15 (65.22) | 1 (4.35) |  |  |
| 3 | 1 (4.35) | 0 (0.00) |  |  |
| **Clinical stage, n (%)** |  |  | -4.052 | <0.001 |
| Ia1 | 0 (0.00) | 6 (26.09) |  |  |
| Ia2 | 0 (0.00) | 2 (8.70) |  |  |
| Ia3 | 0 (0.00) | 3 (13.04) |  |  |
| Ib | 0 (0.00) | 1 (4.35) |  |  |
| IIa | 1 (4.35) | 1 (4.35) |  |  |
| IIb | 4 (17.39) | 9 (39.13) |  |  |
| IIIa | 10 (43.48) | 1 (4.35) |  |  |
| IIIb | 8 (34.78) | 0 (0.00) |  |  |
| IIIc | 0 (0.00) | 0 (0.00) |  |  |
